# Gout Inflammation Time Programming: Molecular Clock from Crystal Triggering to Tissue Remodeling

**DOI:** 10.3390/ijms27031523

**Published:** 2026-02-04

**Authors:** Xin Chen, Chunyuan Zhang, Hanwen Zheng, Qingping Shi, Beiyan Chen, Jieru Han

**Affiliations:** 1 First Clinical Medical College, Heilongjiang University of Chinese Medicine, Harbin 150040, China; radinen@outlook.com (X.C.); 18009333891@163.com (C.Z.); 18282507952@163.com (H.Z.); 2 School of Basic Medical Sciences, Heilongjiang University of Chinese Medicine, Harbin 150040, China; sqp17326705862@163.com (Q.S.); 17323862169@163.com (B.C.)

**Keywords:** gout inflammation, molecular clock, immunometabolic reprogramming, epigenetic memory, stage-specific intervention, digital twin, precision medicine

## Abstract

This review introduces and elaborates a novel temporal paradigm, the “Gout Inflammation Time Programming” model, conceptualized through the Gout-STAT™ framework. This model redefines gout inflammation as a dynamic continuum progressing through three precisely timed phases: an acute Perception phase (0–24 h) initiated by monosodium urate (MSU) crystal recognition, triggering the NOD-like receptor thermal protein domain associated protein 3 (NLRP3) inflammasome and neutrophil-driven burst; a critical Adaptation phase (24–72 h) where outcomes are determined by immunometabolic reprogramming of macrophages and synovial fibroblasts; and a chronic Tissue Injury phase (>72 h) driven by epigenetic memory, leading to irreversible osteoarticular destruction. Deciphering this programmed timeline reveals distinct therapeutic windows. We propose a shift towards stage-specific precision interventions, targeting upstream triggers (e.g., mitochondrial reactive oxygen species(ROS), neutrophil extracellular trap formation (NETosis)) in the acute phase, correcting metabolic checkpoints (e.g., succinate accumulation, impaired autophagy) during adaptation, and employing tissue-protective strategies (e.g., epigenetic modulators) in the chronic phase. Furthermore, we highlight the pivotal role of cutting-edge translational technologies, such as intelligent drug delivery systems and digital twin joint models, in achieving spatiotemporal precision. Understanding this intrinsic molecular clock is fundamental for advancing gout management from reactive treatment to a predictive, preventive, and personalized 4P medicine approach.

## 1. Introduction

Gout, a pervasive inflammatory arthritis, exemplifies a precisely timed pathological program, initiating from the sterile insult of MSU crystal deposition and progressing through defined molecular and cellular stages toward chronic tissue remodeling. It is crucial to distinguish between uric acid and MSU crystals in this context. Soluble uric acid acts primarily as an antioxidant at physiological concentrations, whereas its crystallization into insoluble MSU microcrystals transforms it into a potent ‘danger signal’ or damage-associated molecular pattern. The crystalline structure with its specific surface properties is essential for recognition by pattern recognition receptors. The acute phase is characterized by a cascade of molecular events, beginning with crystal recognition by pattern recognition receptors such as *NLRP3* [1], leading to a mitochondrial ROS burst, *NLRP3* inflammasome assembly, and subsequent caspase-1-dependent maturation of IL-1β [2,3,4,5,6,7]. This cytokine storm orchestrates rapid neutrophil influx, which further amplifies inflammation through phagocytosis, additional cytokine release, and the formation of NETs that entrap crystals but also contribute to the resolution of acute inflammation and the nascent structure of tophi [8,9,10,11]. In addition to the immediate inflammatory cascade, emerging evidence underscores the influence of circadian rhythms and molecular clocks on gout pathogenesis. Gout flares exhibit distinct circadian rhythms and seasonality, potentially governed by the diurnal regulation of enzymes such as xanthine oxidase, seasonal variations in the gut microbiota, and the body’s intrinsic timekeeping systems [12]. This temporal dimension suggests that the host’s inflammatory response is not only triggered by crystals but also programmed by underlying biological rhythms, integrating the “perception” of the crystalline danger signal with a temporally “adapted” immune response. If this programming fails or the inflammatory triggers persist, the process advances to the third stage of “tissue injury,” marked by chronic synovitis, bone erosions, and tophus formation, as visualized by advanced imaging techniques [13,14,15].

While the classic clinical model of gout describes a “flare–intercritical–chronic” continuum based on symptomatic episodes and tophus presence, it offers limited insight into the underlying molecular drivers that govern transitions between these states. The novel “Gout Inflammation Time Programming” model, conceptualized through the Gout-STAT™ framework, advances beyond this descriptive staging by proposing a mechanism-defined, temporal cascade. It reframes progression as a programmed sequence of biological phases—Perception, Adaptation, Tissue Injury—each characterized by dominant cellular players and molecular events on a scale of hours to days. This shift from a clinical to a mechanistic timeline is crucial, as it identifies precise molecular checkpoints and critical transition windows (e.g., the 24–72 h adaptation period) that determine disease fate, thereby providing a rational foundation for stage-specific therapeutic intervention rather than symptom-palliative care.

**Figure 1:**
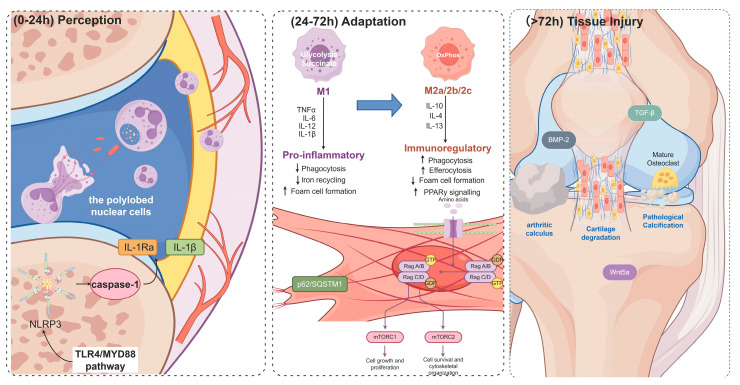
The Gout-STAT™ model: A three-stage temporal continuum of gout inflammation. This schematic illustrates the dynamic progression from the acute Perception phase (0–24 h), characterized by MSU crystal recognition and *NLRP3* inflammasome activation; through the critical Adaptation phase (24–72 h), defined by immunometabolic reprogramming of macrophages and synovial fibroblasts; to the chronic Tissue Injury phase (>72 h), driven by epigenetic memory and leading to irreversible osteoarticular destruction. Solid black arrows indicate primary biological processes or signaling directions. Figure 1 was created with Figdraw (www.figdraw.com).

The Gout-STAT™ model, which conceptualizes this “perception–adaptation–tissue injury” continuum, provides a framework for deconstructing gout’s inflammatory timeline, from the initial molecular clockwork of crystal triggering to the final phase of structural damage, thereby paving the way for stage-specific precision interventions (Figure 1).

## 2. Gout Inflammatory Three-Stage Transformation

### 2.1. Acute Phase (0–24 h): Crystal Trigger and Outbreak Response

During the acute phase (0–24 h) of gout, the deposition of monosodium urate crystals triggers a rapid cascade of molecular and cellular events, leading to an explosive inflammatory response. MSU crystal formation primarily stems from hyperuricemia, where serum uric acid levels exceed the solubility threshold [16]. Crystal precipitation is influenced by multiple factors, including genetic predispositions, diet, and comorbidities [17]. MSU crystals are recognized by immune cells via their specific crystalline faces (e.g., the {011} face), activating pattern recognition receptors such as *NLRP3*, which initiates the inflammatory cascade [2,5,18]. This recognition process involves the TLR4/MYD88 signaling pathway, leading to a burst of mitochondrial ROS and the assembly of the *NLRP3* inflammasome, which activates caspase-1 [2,7,19,20]. Activated caspase-1 cleaves pro-IL-1β and pro-IL-18 into their active forms, leading to the release of IL-1β, a key inflammatory mediator [2,6]. IL-1β recruits neutrophils to the site of crystal deposition, causing the characteristic symptoms of acute gout: pain, swelling, redness, and warmth [2].

In the initial recognition stage (<2 h), the physicochemical properties of MSU crystals are coated with apolipoproteins, such as differences in surface charge distribution and the formation of a “protein corona”. This corona, which includes adsorbed complement C3b and IgG, modulates the interaction between crystals and immune cells [21,22]. Tissue-resident macrophages phagocytose crystals via a spleen tyrosine kinase(SYK)-dependent pathway, leading to phagosomal rupture and amplification of inflammatory signaling, a key process known to activate the NALP3 inflammasome [9,23,24]. Concurrently, mast cells may release histamine via receptors (e.g., Mas-related G protein-coupled receptor member X2(MRGPRX2)), exacerbating the early inflammatory response [25]. The overall inflammatory process in gout is also influenced by systemic factors, including alterations in the gut microbiota [26], which plays a fundamental role in immune regulation [27]. Future investigations employing advanced techniques such as single-cell transcriptomics [28] will further delineate the cellular interactions underlying this pathology.

**Figure 2:**
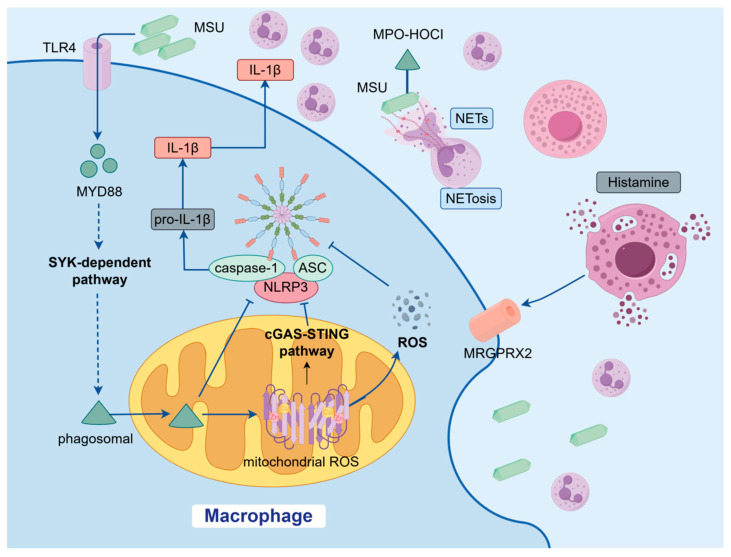
Molecular cascade in the acute phase of gout. Upon deposition, MSU crystals are recognized by pattern recognition receptors (e.g., TLR4), leading to mitochondrial reactive oxygen species (ROS) burst, *NLRP3* inflammasome assembly, and caspase-1-dependent maturation of IL-1β. This key cytokine orchestrates a rapid neutrophil influx, which amplifies inflammation through phagocytosis, additional cytokine release, and the formation of neutrophil extracellular traps (NETs). Solid line arrow indicate direct activation, promotion, or transformation. T-bar/Blunt arrow represent inhibition, blockade, or negative regulation. Dashed line arrow denote indirect effects, modulation, or potential influences. Figure 2 was created with Figdraw (www.figdraw.com).

As inflammation progresses (2–11 h), an inflammatory amplifier forms, centered around the mitochondrial ROS–NLRP3 axis. Crystals induce mitochondrial DNA oxidation, synergistically activating the STING pathway and promoting the spatial organization of ASC speck formation, thereby optimizing inflammasome assembly [2,7]. Furthermore, oxidation of components within the adsorbed protein corona, such as apolipoproteins, can further amplify the pro-inflammatory signaling triggered by MSU crystals. Massive neutrophil infiltration leads to NETosis, where NETs release DNA and histones, transporting IL-1β deeper into tissues and etching the crystal surface via MPO-HOCl complexes, thereby increasing their proinflammatory potential [9,10,11]. Beyond their classic pro-inflammatory role, infiltrating neutrophils also undergo a metabolic reprogramming, shifting toward glycolysis to fuel their effector functions. Importantly, as part of a self-limiting response, these cells can contribute to inflammation resolution through the production of specialized pro-resolving mediators (SPMs) and by undergoing phenotypic switches that help clear cellular debris and apoptotic neutrophils. This functional plasticity is exemplified by their concurrent release of both pro-inflammatory IL-1β and its natural inhibitor, interleukin-1 receptor antagonist (IL-1Ra), which begins to buffer the cytokine storm. Furthermore, a subset of neutrophils undergoes a metabolic and secretory switch to produce anti-proteases and pro-resolving lipid mediators, such as resolvins and protectins, initiating the earliest molecular cues for inflammation resolution. This cascade not only amplifies local inflammation but also promotes persistent crystal deposition, potentially leading to tophus formation (Figure 2). Tophi typically form within or around joints, including subchondral bone areas, bursae, and tendon sheaths. Joint aspiration often reveals white, chalk-like material suspended in synovial fluid, and tophi can erode underlying bone, creating characteristic radiologic “punched-out” lytic lesions with overhanging edges [15,29,30]. However, the outcome of this acute inflammatory storm—whether it resolves swiftly or progresses to chronicity—is not decided in the first 24 h, but in the subsequent critical time window defined by profound immunometabolic reprogramming within the joint microenvironment [31].

### 2.2. Transition Phase (24–72 h): Immunometabolic Reprogramming

The transition phase of gout (24–72 h) represents a decisive crossroads in the disease’s temporal program. Following the acute burst, the transition phase of gout (24–72 h) represents a critical period during which the resolution of acute inflammation is determined and is largely governed by intricate immunometabolic reprogramming [31]. The transition from neutrophil-dominated acute inflammation to macrophage-mediated resolution is a coordinated process. Neutrophil-derived SPMs and apoptotic neutrophils themselves provide critical signals that promote the phenotypic switch of macrophages from a pro-inflammatory M1 to an anti-inflammatory M2 state. A key pathophysiological feature is the impaired phenotypic switch of macrophages. During an acute attack, proinflammatory M1 macrophages dominate the scene and are responsible for the massive release of IL-1β. These cells are characterized by a metabolic shift toward enhanced glycolysis, a process potentiated by the inactivation of the mitochondrial pyruvate carrier, which skews energy production toward glycolysis [19]. The spontaneous remission of acute gouty inflammation is associated with a transition to anti-inflammatory M2 macrophages [32,33], a process that can be modulated by factors such as melatonin, which promotes a metabolic shift from glycolysis to oxidative phosphorylation [9,25,34]. However, failure to resolve inflammation is often due to a metabolic blockade that prevents this M1-to-M2 transition, thereby perpetuating an inflammatory milieu that sustains neutrophil recruitment and activity. The persistence of M1 metabolism may be driven by two key metabolic disruptions: succinate accumulation, as evidenced by metabolomic studies identifying dysregulated pathways in gout progression [30,35,36,37], which can inhibit histone demethylases and NAD+ depletion, potentially linked to dysregulated nicotinate and nicotinamide metabolism [37]. These disruptions lead to SIRT1 inactivation and sustained NF-κB pathway activity, thereby maintaining a proinflammatory gene expression profile. Concurrently, butyrate, through the inhibition of class I HDACs [38], exerts a potent anti-inflammatory effect by suppressing cytokine production, highlighting the importance of epigenetic regulation in this process [4,39]. Furthermore, the upregulation of CD39, which promotes remission by hydrolyzing proinflammatory extracellular ATP, underscores the active metabolic reprogramming required for inflammation resolution [25,40]. This functional inflexibility, rooted in metabolic-epigenetic crosstalk, effectively locks macrophages in a pro-inflammatory state, disrupting the clearance of apoptotic cells and perpetuating neutrophil recruitment.

Parallel to macrophage dysfunction, the malignant transformation of synovial stromal cells occurs. Synovial fibroblasts (also known as fibroblast-like synoviocytes, FLSs) undergo metabolic reprogramming, resulting in marked enhancement of the Warburg effect [41]. This is evidenced by a significant upregulation of glucose transporters, leading to excessive glycolysis and lactic acid accumulation, which may be reflected in the distinct serum metabolomic and lipidomic profiles of gout patients [37,42,43]. The resulting decrease in local pH can activate ASICs, further perpetuating inflammatory and pain signals [2,43]. Additionally, there is a disruption in autophagic flux within these cells. The accumulation of p62/SQSTM1, a selective autophagy substrate, can aberrantly activate the mechanistic target of rapamycin complex 1 (mTORC1) pathway. This activation promotes the secretion of profibrotic factors, paving the way for chronic joint damage and tophus formation, which can be visualized through advanced imaging techniques [44,45,46,47,48].

**Figure 3:**
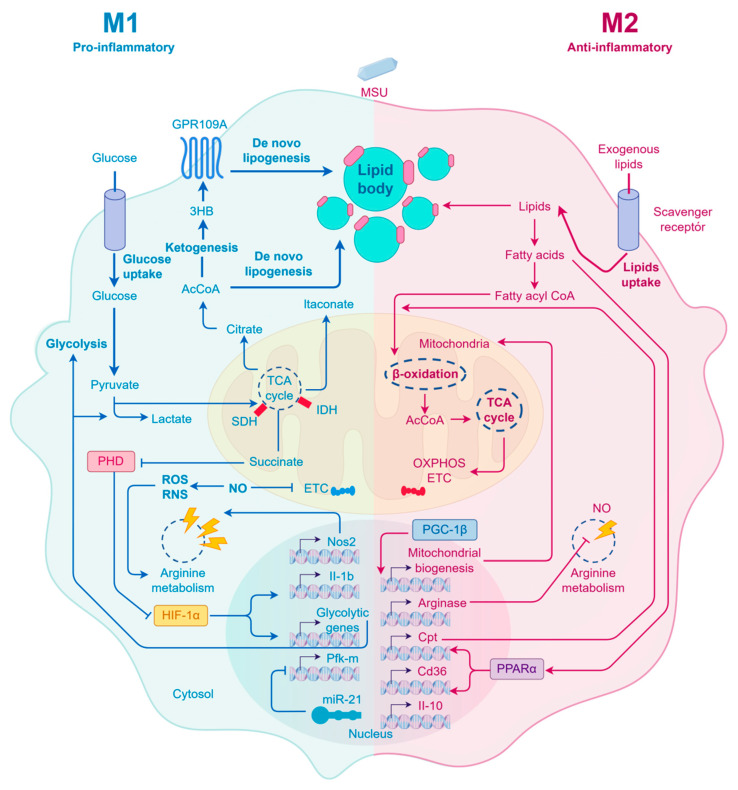
Immunometabolic reprogramming dictates the fate of inflammation during the transition phase. Failure to resolve inflammation is often due to a metabolic blockade that prevents the M1-to-M2 macrophage transition. Proinflammatory M1 macrophages exhibit enhanced glycolysis and succinate accumulation, whereas anti-inflammatory M2 macrophages rely on oxidative phosphorylation. Therapeutic interventions (e.g., metformin, SUCNR1 antagonists) aim to reverse this aberrant metabolic state to promote resolution. Solid line arrow indicate direct activation, promotion, or transformation. T-bar/Blunt arrow represent inhibition, blockade, or negative regulation. Figure 3 was created with Figdraw (www.figdraw.com).

This maladaptive response in FLS, coupled with dysfunctional macrophage metabolism, creates a vicious cycle that impedes the resolution of inflammation and facilitates the transition of gout from an acute, self-limiting condition to a chronic, destructive arthropathy. Predictive models and biomarkers, such as the Gout Activity Score (GAS) and serum HDL-C levels, are being developed to better identify and manage patients in this critical transition phase [49,50] (Figure 3). If this adaptive reprogramming fails, the maladaptive responses in macrophages and synovial fibroblasts become entrenched, creating a self-sustaining vicious cycle that propels the disease inexorably toward the chronic phase characterized by tissue memory and structural damage [51].

### 2.3. Chronic Phase (>72 h): Tissue Memory Formation

**Figure 4:**
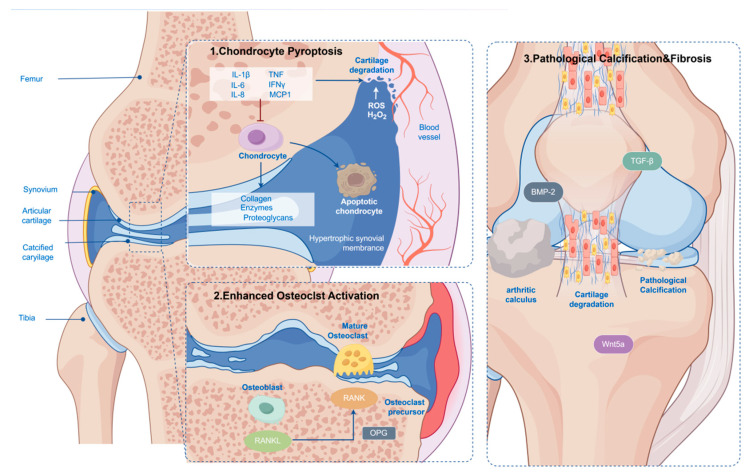
The triad of osteoarticular destruction in chronic gout. Persistent inflammation and epigenetic memory drive irreversible damage across joint tissues: (**1**) Cartilage degradation via chondrocyte pyroptosis and matrix degradation; (**2**) Bone erosion due to enhanced osteoclast activation mediated by the RANKL/RANK/OPG system; (**3**) Pathological calcification and fibrosis in tendons and ligaments, facilitated by factors such as BMP-2 and Wnt5a. Solid line arrow indicate direct activation, promotion, or transformation. T-bar/Blunt arrow represent inhibition, blockade, or negative regulation Figure 4 was created with Figdraw (www.figdraw.com).

When inflammation fails to resolve effectively during the transition phase, recurrent inflammatory episodes lead to the chronic phase (>72 h), characterized by the establishment of persistent tissue memory, with epigenetic imprinting as a core mechanism [51]. This memory is not only retained within immune cells but also profoundly impacts joint tissue homeostasis, ultimately manifesting as an irreversible triad of osteoarticular destruction (Figure 4 and Table 1). Research indicates that uric acid can mechanistically alter the inflammatory capacity of myeloid cells through the induction of transcriptional and epigenetic reprogramming, a process termed “urate-induced immune programming,” which forms the foundation of tissue memory in gout [52,53]. This reprogramming likely involves alterations in chromatin accessibility and hyperactivation of inflammatory enhancers, enabling a more rapid and robust response of inflammation-related genes upon subsequent stimulation. This concept aligns with the phenomenon of trained immunity, where innate immune cells undergo long-term functional reprogramming after initial exposure to stimuli such as MSU crystals, leading to an exaggerated inflammatory response upon rechallenge [51,54].

This long-term tissue memory and chronic inflammation ultimately manifests as an irreversible triad of osteoarticular destruction. Within cartilage, the persistent inflammatory microenvironment, particularly driven by continued *NLRP3* inflammasome activation and IL-1β release [55], can promote chondrocyte pyroptosis and exacerbate matrix degradation. Imaging studies have confirmed the prevalence of cartilage erosion and bone erosion in the joints of chronic gout patients [30,45,56,57,58]. Bone destruction is driven by aberrant osteoclast activation, whereas pathological calcification or fibrosis can occur in tendons and other soft tissues. These processes collectively contribute to the joint deformity and functional loss characteristic of chronic gout. Notably, a dysfunctional missense variant in the *NUMB* gene undergoes intracellular redistribution and degradation via an autophagy-dependent mechanism, impairing the membrane localization of the urate transporter *ABCG2*. This ultimately leads to defective uric acid excretion and hyperuricemia, providing a novel perspective on the interplay between genetic and epigenetic factors in chronic gout [59,60].

The mechanisms underlying tissue memory formation in the chronic phase can be further detailed as follows:

#### 2.3.1. Epigenetic Memory Formation

Histone modifications: Persistent inflammatory signaling may cause lasting alterations in histone modifications at the promoters of key proinflammatory genes (e.g., TNF, IL-6), such as the sustained deposition of H3K4me3 and hypersensitization of H3K27ac, thereby maintaining the transcriptional activity of these genes. These modifications are closely associated with consistently elevated levels of inflammatory mediators such as ox-LDL, hs-CRP, IL-6, and TNF-α, which are observed in chronic inflammation [61,62,63].

Altered Chromatin Spatial Architecture: Chronic inflammation may induce a reorganization of the three-dimensional genome architecture, particularly the aberrant strengthening of enhancer-promoter interactions within TADs containing inflammation-related gene loci, thereby cementing the proinflammatory phenotype [52,63,64]. Such alterations in spatial conformation might underlie the sustained high expression of key transcription factors such as *NFIL3* in neutrophils from gout patients, which subsequently promotes autophagy and NET formation via the *REDD1*/*mTOR* axis, exacerbating inflammation [22].

#### 2.3.2. Triad of Osteoarticular Destruction

This persistent low-grade inflammation and structural damage, driven by epigenetic memory, underscores the complexity of managing chronic gout. Therapeutic approaches must evolve beyond traditional strategies focused solely on acute-phase anti-inflammatories, such as diclofenac or IL-1 inhibitors, and urate-lowering agents, such as allopurinol, febuxostat, or lesinurad, as supported by references respectively [49,50,65,66]. Future directions should explore novel therapies capable of intervening in epigenetic programming and promoting tissue repair. Long-term management strategies should adhere to the Treat-to-Target principle, aiming to eliminate urate crystals, prevent flares, reverse chronic damage [67], and utilize advanced imaging techniques such as ultrasound and dual-energy computed tomography (DECT) to monitor treatment response [15,29,68].

**Table 1:** Triad of osteoarticular destruction.

Target Tissue	Core Mechanism	Key Effector Molecules/Pathways	Clinical/Imaging Correlation
Cartilage	Chondrocyte Pyroptosis and Matrix Degradation	Gasdermin D, Various Matrix Degrading Enzymes	Double contour sign on US; Cartilage erosion on MRI [30,37,57]
Bone	Enhanced Osteoclast Activation	*RANKL/RANK/OPG* System	Bone erosions and bone marrow edema detectable by DECT/MRI [30,44,69]
Tendons/Ligaments	Pathological Calcification, Fibrosis and Tophus Formation	BMP-2, Wnt5a, TGF-β	DECT allows volume quantification of urate deposits; US visualizes tophi and aggregates [29,37,58]
Target Tissue	Core Mechanism	Key Effector Molecules/Pathways	Clinical/Imaging Correlation

This table summarizes the core mechanisms, key molecular pathways, and corresponding clinical/imaging correlates of the three primary tissue injuries in chronic gout, highlighting the multifaceted nature of joint destruction.

## 3. Treatment Transformation

### 3.1. Phase-Specific Interventions

#### 3.1.1. Targeted Intervention in the Acute Phase (<24 h)

The management of acute gout has evolved to include targeted interventions aimed at specific molecular pathways activated during the initial 24 h inflammatory burst. These strategies seek to interrupt the core inflammatory cascade at its source, potentially offering faster and more specific relief with fewer side effects than conventional anti-inflammatory agents do.

A promising therapeutic avenue involves the use of mitochondrial ROS scavengers. The central role of the mitochondrial ROS–NLRP3 axis in initiating inflammasome assembly and IL-1β maturation makes it a prime target [2,9,70,71]. Compounds such as MitoQ, a coenzyme Q10 derivative that accumulates within mitochondria, are designed to neutralize this initial oxidative burst [72,73]. By quenching mitochondrial ROS, MitoQ effectively blocks the activation of the *NLRP3* inflammasome, preventing the downstream cascade that leads to neutrophil recruitment and the characteristic severe pain and swelling [74,75,76].

Moreover, the inhibition of NETosis has emerged as another strategic intervention. NETs play a critical role in amplifying and sustaining inflammation in acute gout by delivering IL-1β deep into tissues and modifying MSU crystals to increase their proinflammatory potential [9]. By targeting this process, peptidylarginine deiminase 4 (PAD4) inhibitors such as GSK484 prevent the citrullination of histones, a key step in the formation and release of NETs [38]. By inhibiting NETosis, these compounds can dampen the feed-forward inflammatory loop without completely compromising neutrophil-based host defense. Preclinical studies, including those in primate models, have shown efficacy in reducing inflammation, suggesting that PAD4 inhibition could be a viable strategy to control the destructive inflammatory amplification driven by neutrophils in the acute phase [9,77,78].

These targeted approaches represent a shift toward precision medicine in acute gout management, moving beyond broad-spectrum immunosuppression to directly address the key molecular and cellular events that define the first 24 h of a gout flare [79,80] (Table 2).

#### 3.1.2. Key Regulatory Mechanisms During the Transformation Period (24–72 h)

On the basis of an in-depth understanding of the immunometabolic reprogramming mechanisms during the gout transition phase (24–72 h), targeted intervention strategies show significant therapeutic potential. In terms of metabolic reprogramming in macrophages, targeting their aberrant glycolytic pathway is crucial. For example, metformin, an AMP-activated protein kinase (AMPK) activator, can reverse the glycolysis process upon which M1 macrophages depend. This mechanism functionally counteracts the glycolytic reprogramming and *NLRP3* inflammasome activation caused by MPC inhibition [19,81,82]. By shifting macrophage metabolism from proinflammatory glycolysis back toward oxidative phosphorylation, such drugs are expected to promote inflammation resolution. Similarly, MLT-MLP, has also been shown to remodel macrophage metabolism from glycolysis to oxidative phosphorylation, thereby effectively alleviating acute gouty arthritis [25,37], providing another strong rationale for metabolic intervention. Furthermore, metabolomic studies not only suggest the accumulation of metabolites such as succinate during gout progression [30,37] but also reveal significant dysregulation of multiple pathways, including histidine, nicotinate, and nicotinamide metabolism, during disease progression. These findings provide a solid theoretical basis for the use of SUCNR1 antagonists, which aim to block succinate-mediated sustained inflammatory signaling and epigenetic reprogramming, thereby erasing macrophage “inflammatory memory.”

On the other hand, autophagy activators that target synovial stromal cells are also essential. During the transition phase, blocked autophagy flux leads to p62/SQSTM1 accumulation, which aberrantly activates the mTORC1 signaling pathway and promotes fibrosis [19]. Therefore, the application of autophagy inducers such as the Tat-Beclin1 peptide can effectively clear accumulated p62 and restore autophagic function, thereby inhibiting the secretion of mTORC1-driven profibrotic factors. This strategy mechanistically targets one of the core pathological links in chronic gout, namely, the tissue remodeling and chronic joint damage caused by the malignant transformation of stromal cells such as synovial fibroblasts [48,61,83]. Interfering in the autophagy–metabolism axis can significantly alleviate synovial fibrosis and tophus formation, altering the chronic progression trajectory of the disease (Table 3).

#### 3.1.3. Chronic Phase Tissue Protection (>72 h)

In the chronic phase of gout (>72 h), therapeutic strategies are increasingly focused on tissue protection to counteract the irreversible damage driven by persistent inflammation and established tissue memory [52,84]. Targeting the underlying epigenetic dysregulation and aberrant cellular differentiation processes holds significant promise for preventing the structural joint damage characteristic of advanced gout [30,45].

Epigenetic modulators represent a novel class of therapeutic agents aimed at resetting the pathological gene expression programs that sustain chronic inflammation. Bromodomain and extraterminal (BET) protein inhibitors, such as JQ1, can disrupt the reading of acetylated histone marks, thereby preventing the recruitment of the transcriptional machinery to key inflammatory gene promoters. This action can effectively “reset” the proinflammatory chromatin state that characterizes chronic gout and is sustained by mechanisms such as urate-induced immune programming [52,85,86]. The potential for such epigenetic interventions is supported by evidence of altered methylation patterns in gout, such as elevated UMOD methylation in peripheral blood [20,87], demonstrating that the gouty state is associated with stable epigenetic alterations. By modulating the epigenetic landscape, these inhibitors offer a strategy to disrupt the chronic inflammatory cycle and mitigate long-term tissue damage, potentially addressing the persistent elevation of inflammatory mediators such as IL-6 and TNF-α observed in gout patients.

Concurrently, protecting mesenchymal stem cells within the joint from pathological signaling is crucial to prevent the triad of osteoarticular destruction. In tendons, aberrant activation of the noncanonical Wnt pathway, particularly through *Wnt5a* overexpression, can drive tendon-derived stem cells toward abnormal osteogenic differentiation, contributing to ectopic bone formation and enthesopathy [87]. This pathogenic process is part of a broader dysregulation of tissue homeostasis in the chronic inflammatory environment [88,89]. Anti-Wnt5a monoclonal antibodies have demonstrated efficacy in preclinical models by specifically blocking this pathogenic signal, thereby reducing osteophyte formation [90]. This approach directly addresses one arm of the destructive triad of chronic gouty arthropathy by preventing structural damage at its source [90,91]. The efficacy of such targeted biologics could be monitored via advanced imaging techniques such as DECT, which allows the quantification of urate volume, and MRI, which can detect associated soft tissue and bone pathology [9,15].

The integration of these tissue-protective strategies aligns with the evolving treat-to-target approach in gout management [67], which aims not only to control serum urate levels but also to prevent long-term joint damage. The dysfunctional *NUMB*/*ABCG2* pathway, which impairs urate excretion and contributes to chronic hyperuricemia [59], may also interact with these pro-fibrotic and pro-calcific signaling pathways, suggesting potential synergistic therapeutic targets. Combining epigenetic modifiers that reverse inflammatory memory with biologic agents that protect stem cells could represent a next-generation strategy for chronic-phase tissue protection in gout (Table 4). This strategy aims for true disease modification and joint preservation, moving significantly beyond the scope of traditional urate-lowering drugs like allopurinol or febuxostat [49,50]. This is particularly important given the challenges of medication adherence in chronic gout management [92], where therapies that provide tangible protection against structural damage may improve long-term patient engagement [89,93,94].

### 3.2. Frontier Technology

Achieving “phase-specific intervention” in gout inflammation urgently requires the support of cutting-edge technologies, among which intelligent responsive drug delivery systems and digital twin joint models represent the most promising directions. Intelligent drug delivery systems aim to deliver drugs to the lesion site with precise dosages and at the correct time. ROS-responsive hydrogels are a prime example, designed to leverage the characteristic high levels of reactive oxygen species in the gout inflammatory microenvironment, a feature particularly prominent during the acute and transition phases. These hydrogels are composed of polymers containing thioketal bonds or selenium, which are cleaved in high-ROS environments, leading to hydrogel degradation and the release of encapsulated drugs, such as IL-1β inhibitors [95]. This strategy can significantly increase the local drug concentration in the joint cavity while reducing the risk of immunosuppression associated with systemic administration. Its effectiveness has been validated in preclinical models, which have shown significant shortening of the acute inflammatory phase and prevention of chronic synovial thickening compared with conventional injections [2,9,96]. Another innovation is the use of macrophage membrane-coated nanoparticles, which utilize cell membranes extracted from M2 macrophages to coat drug-loaded nanoparticles. By leveraging targeting molecules retained on the membrane, these nanoparticles actively home to inflamed joints, are phagocytosed by local macrophages, and release the drug upon cleavage in specific intracellular environments (e.g., low pH or high ROS), achieving “intracellular targeted therapy,” for instance, by delivering SIRT1 activators to regulate cell metabolism and phenotype+.

**Figure 5:**
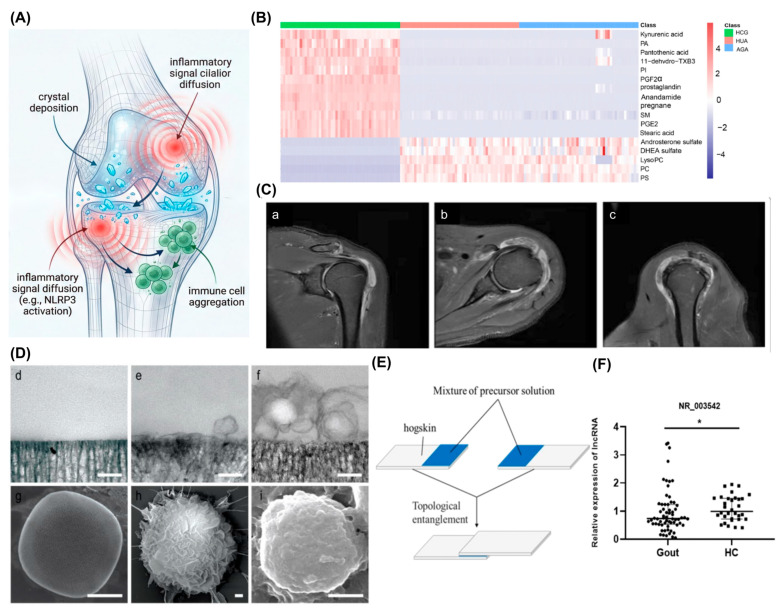
Cutting-edge technologies for spatiotemporal precision medicine in gout. (**A**) The digital twin joint model integrates multi-omics and clinical data to construct a patient-specific virtual joint, enabling dynamic simulation of disease progression and in silico prediction of optimal therapeutic strategies. (**B**) Metabolomics data from [98] with permission from BioMed Central, copyright 2023. (**C**) Shoulder joint MRI of a 63-year-old male gout patient showing no obvious soft-tissue invasion around tophi: (**a**) T2-FS coronal, (**b**) T2-FS transverse, (**c**) T2-FS sagittal. From [100] with permission from BioMed Central, copyright 2022. (**D**) Macrophage membrane-coated nanoparticles (M2-membrane-camouflaged). (**d**) Transmission electron micrograph (TEM) of bare porous nanoparticles. (**e**,**f**) Coating thickness varies with the number of membrane layers and their fusion extent. (**g**–**i**) Scanning electron micrograph (SEM) of bare nanoparticles (**g**), a leukocyte membrane donor (**h**), and a membrane-camouflaged nanoparticle (**i**). From [103] with permission from Springer Nature, copyright 2013. (**E**) ROS-responsive hydrogel for targeted drug release at inflamed joints. From [96] with permission from BioMed Central, copyright 2021. (**F**) Transcriptomic data from [101] with permission from Public Library of Science, copyright 2021. In the graphs, asterisks indicate statistical significance: * *p* < 0.05.

Another revolutionary tool breaking the current “one-size-fits-all” treatment model is the digital twin joint model. This model constructs an individualized virtual joint by integrating patient multiomics data—including genomic *ABCG2* mutation status [97], transcriptomic inflammatory signaling profiles, metabolomic serum metabolites [98], and radiomic data such as urate volume from dual-energy CT [99] and bone erosion scores from MRI [100]—with clinical information. Using mechanical models and deep learning algorithms, the model can dynamically simulate the disease process, including crystal deposition, inflammatory signal cascade amplification as exemplified by *NLRP3* activation [2,13], immune cell recruitment as seen in NETosis [14], and tissue repair. In clinical translation applications, the model can output an individual’s “chronicity risk index” and “predicted transition time window,” and perform treatment regimen sandbox simulations in virtual space. For example, the long-term effects of using NETosis inhibitors during an acute flare can be compared with those of metabolic reprogramming drugs, such as agents targeting the succinate pathway, during the transition window [100,101,102] (Figure 5).

Together, these cutting-edge technologies provide a new blueprint for the future diagnosis and treatment of gout: shifting from passive reactions to active programming. By deciphering the inherent inflammatory processes and biological rhythms of the disease [12], utilizing intelligent drug delivery systems for spatiotemporally precise intervention, and combining the individualized navigation provided by digital twin models, gout treatment is moving toward a new era of 4P medicine characterized by “prediction, prevention, personalization, and participation.” Future challenges lie in the clinical translation of these technologies, cost optimization, and the establishment of ethical and regulatory frameworks, but their potential is undoubtedly a transformative force capable of rewriting the disease trajectory for millions of gout patients [100,102,104,105].

While intelligent drug delivery systems and digital twin models represent transformative approaches, their path to clinical adoption is accompanied by significant challenges. ROS-responsive hydrogels, though promising in preclinical models, face hurdles related to biocompatibility, long-term safety, scalable manufacturing, and regulatory approval for intra-articular use [95,96,104]. Their responsiveness must be finely tuned to avoid off-target release, and large-scale clinical trials are needed to validate efficacy and safety in human gout patients. Similarly, digital twin joint models, while powerful in silico tools, require robust validation against real-world clinical outcomes. Challenges include data integration from heterogeneous sources (genomic, imaging, metabolomic), model transparency and interpretability, computational resource demands, and ethical considerations regarding data privacy [102,106,107]. Moreover, the high cost of developing and maintaining such models may limit accessibility in routine clinical settings [108,109]. Future work must therefore focus not only on technological refinement but also on health economic evaluations, stakeholder engagement, and the development of regulatory frameworks to facilitate the translation of these precision medicine tools into actionable clinical strategies [110,111,112].

## 4. Disputes and Prospects

### 4.1. The Unsolved Mystery: The Underlying Puzzle Revealed by the Model

#### 4.1.1. The Mystery of Disease Resilience: Why Do Some Patients Not Progress to Chronicity

Approximately 20% of gout patients remain in the acute attack stage throughout their lives, demonstrating significant disease resilience. This resilience may stem from a complex protective network involving genetic, epigenetic, and metabolic factors (Table 5).

Genome-wide association studies have revealed that certain loss-of-function variants in the *CARD8* gene are closely associated with a weakened inflammatory response. These variants may provide strong protection to carriers by disrupting the stable assembly of the inflammasome [113,114]. Additionally, single-nucleotide polymorphisms in the regulatory regions of *IL1RN* may elevate baseline levels of the IL-1 receptor antagonist, enhancing negative feedback on IL-1 signaling. Genetic variation in urate transporters such as *ABCG2* and *SLC2A9* also influences gout susceptibility and may contribute to resilience by promoting efficient urate excretion and reducing intracellular crystal load [97,114,115].

We hypothesize that immune cells of resilient individuals may possess an “epigenetic buffering” mechanism. For example, the promoter regions of key proinflammatory genes (such as *IL-1β* and *TNF*) might maintain more stable repressive histone marks [116] (e.g., H3K27me3), thereby resisting excessive activation triggered by inflammatory signals. The anti-inflammatory effect of butyrate via the inhibition of class I HDACs demonstrates the potential role of epigenetic regulation in modulating gout inflammation [38,39,117]. Non-coding RNAs, including miR-146a, may also fine-tune inflammatory responses by targeting downstream signaling adaptors (e.g., IRAK1, TRAF6).

Macrophages from resilient individuals might exhibit greater metabolic flexibility, enabling a rapid switch from glycolysis to oxidative phosphorylation. This prevents succinate accumulation and subsequent HIF-1α-driven epigenetic dysregulation, thereby promoting timely resolution of inflammation [35,36,61]. Recent serum metabolomic profiling of non-progressors supports this notion, showing lower succinate/α-ketoglutarate ratios and distinct glycolytic intermediate patterns compared to progressors [98,118]. The role of the gut microbiota and metabolites such as butyrate in influencing host metabolism and inflammation further supports the link between the metabolic state and the gout phenotype The gut microbiota and its metabolites, particularly short-chain fatty acids such as butyrate, may further reinforce resilience by systemically modulating host immune-cell metabolism and epigenetic landscapes [119,120,121,122].

Identifying these “resilience factors” holds immense translational value. Future research should prioritize longitudinal studies integrating genetic, epigenetic, metabolomic, and microbiomic data to define resilient subgroups and uncover the molecular basis of their protection. Such insights could guide the development of therapies that mimic natural protective states—for instance, small molecules that stabilize *CARD8*, epigenetic editors that enforce repressive chromatin signatures, or metabolic modulators that enhance oxidative phosphorylation in macrophages [31,114,115,123].

**Table 5:** Potential Protective Mechanisms Underlying Disease Resilience in Gout.

Mechanism Category	Key Factors/Pathways	Proposed Protective Role	Research Status/Evidence Level
Genetic Buffering	*CARD8* loss-of-function variants (e.g., p.C10X)	Disrupts stable NLRP3 inflammasome assembly, reducing IL-1β release.	GWAS-validated; functional studies support [113,114].
	*IL1RN* promoter SNPs	May increase baseline IL-1Ra expression, enhancing feedback inhibition of IL-1 signaling.	Association studies; mechanistic plausibility.
	*ABCG2*/*SLC2A9* high-function variants	Promotes urate excretion, reducing intracellular urate load and crystallization risk.	Validated in multiple populations; functionally established [97,114].
Epigenetic Buffering	Enriched H3K27me3/reduced H3K4me3 at pro-inflammatory gene promoters	Maintains repressive chromatin state, resisting excessive activation by inflammatory signals.	Inferred from epigenomic concepts; analogy to other diseases [116].
	Non-coding RNAs (e.g., miR-146a) upregulation	Targets and inhibits signaling nodes (e.g., IRAK1, TRAF6), providing negative feedback.	Preliminary detection in gout; established in RA.
	Butyrate via HDAC inhibition	Enhances anti-inflammatory gene expression and suppresses pro-inflammatory cytokines.	Supported byin vitroexperiments [38,39,117].
Metabolic Buffering	Macrophage metabolic plasticity (rapid glycolysis → OXPHOS switch)	Prevents succinate accumulation, HIF-1α stabilization, and epigenetic dysregulation, favoring timely resolution.	Supported by metabolic flux analyses; pending validation in gout [35,36,61].
	Lower serum succinate/α-ketoglutarate ratio	Reflects TCA cycle integrity; may inversely correlate with chronicity risk.	Preliminary metabolomic cohort findings [98,118].
Systemic Buffering	Gut microbiota-derived butyrate	Promotes Treg differentiation via GPR109A/PPARγ, enhancing immune tolerance.	Animal model support; associative human data [119,120,121,122].
	Microbiota-immune axis homeostasis	Maintains systemic inflammatory threshold, reducing risk of excessive sterile inflammation.	Conceptual support; mechanisms require elucidation.

This table summarizes potential genetic, epigenetic, metabolic, and systemic factors that may confer resilience against gout progression, their hypothesized protective roles, and current evidence levels. These factors likely interact within a “resilience network” to prevent the transition to chronic disease.

#### 4.1.2. Gut–Joint Axis: What Is the Precise Mechanism of Remote Regulation

The gut microbiota is established as an independent risk factor for gout, yet the precise molecular pathways through which it remotely regulates joint inflammation constitute a critical “black box” in our understanding. Moving beyond correlation, recent research points to several specific mechanistic routes:

1. Metabolite-Mediated Communication: Gut microbiota-derived metabolites act as crucial systemic messengers. A key pathway involves short-chain fatty acids (SCFAs). Reduced levels of anti-inflammatory SCFAs (e.g., butyrate) in gout patients may weaken their inhibitory effects on peripheral immune cells. Butyrate has been shown to suppress MSU crystal-induced cytokine production in human monocytes via inhibition of class I histone deacetylases (HDACs) [2,39]. Conversely, other microbial metabolites may exert pro-inflammatory effects. For instance, succinate, which can be produced by certain gut bacteria, may reach the joint and exacerbate inflammation via the SUCNR1 receptor on macrophages, linking gut metabolism to in situ immunometabolic reprogramming [36,118]. Furthermore, the gut microbiome contributes to the host’s purine and uric acid pool through the metabolism of dietary nucleotides and the expression of microbial uricase, directly influencing hyperuricemia [124,125].
2. Systemic Immune Cell Training: Microbial components or metabolites may systemically “train” innate immune cells, altering their baseline state and subsequent response to MSU crystals. This aligns with the broader concept of “trained immunity” [51]. For example, systemic exposure to microbial ligands may prime bone marrow myeloid precursors, leading to neutrophils with a heightened propensity for NETosis or monocytes/macrophages with a lower activation threshold upon encountering crystals [9,126]. This could explain the “hyper-responsive” phenotype observed in some gout patients.
3. Barrier Integrity and Antigenic Mimicry: Intestinal dysbiosis is often associated with increased gut permeability (“leaky gut”). This may facilitate the translocation of microbial products (e.g., lipopolysaccharide, LPS) into the circulation, contributing to a low-grade systemic inflammatory state that could lower the threshold for acute gout flares [127]. A more speculative hypothesis involves molecular mimicry, where immune responses primed against gut microbial antigens cross-react with structurally similar components in joint tissues, potentially contributing to chronic synovitis [110].
4. Future Imperative: Disentangling this complex axis requires an integrated multi-omics approach. Future studies should concurrently analyze the gut metagenome, serum metabolome (particularly microbial metabolites), and synovial immunome from the same patients across disease phases to move from association to causal understanding [51,106,118].

### 4.2. Future Directions: TOWARD Precision Prevention and Treatment

Future research on gout is progressively shifting from a traditional “one-size-fits-all” model to a precision medicine paradigm [110], with its core focus being the realization of individualized prevention and treatment through the integration of dynamic, multidimensional data. This transition is manifested primarily in two key directions:

#### 4.2.1. Spatiotemporal Multi-Omics Integration Analysis

Current research often relies on static biological samples, making it difficult to capture the dynamic progression of gouty inflammation. Future mechanistic studies must adopt a “dynamic movie” style design, in which patient blood and synovial fluid samples are systematically collected at precise time points during a gout attack (e.g., 0 h, 12 h, 48 h, and 7 d). Single-cell multiomics sequencing can simultaneously resolve the transcriptomic and epigenomic information of the same cell, thereby directly revealing the gene regulatory networks driving inflammatory cell state transitions, such as the role of histone modifications (e.g., H3K4me3) in macrophage polarization [113,123,124]. Concurrent spatial transcriptomics and proteomics technologies can enable in situ observations of the propagation patterns of inflammatory signals within microenvironments such as the synovium, cartilage, and bone, identifying “inflammatory hubs” and their spatiotemporal relationships with urate crystal deposition [106,107,111,128]. The goal of integrating these technologies is to construct the world’s first “Spatiotemporal Atlas of Gout Inflammation” to define the molecular tipping points in the transition from acute inflammation to chronic fibrosis and to identify critical intervention windows and targets [129,130]. For example, this atlas would clarify key dynamic balances, such as that between pro-inflammatory IL-1β and its endogenous inhibitor IL-1Ra, as well as the interplay between TGF-β1 and IL-1β [2,131].

#### 4.2.2. Precision Stratification in Clinical Trial Enrollment

The frequent failure of current clinical trials can be partly attributed to the high heterogeneity of the patient population being treated as a single entity [112]. Future trial designs urgently need to evolve from “clinical staging” to “molecular phenotyping,” refining enrollment criteria into subtypes such as the “Acute Inflammatory Phenotype” defined by elevated baseline levels of inflammatory cytokines such as IL-1βand IL-6 [132]; the “Metabolic Dysregulation Phenotype” characterized by serum metabolomic profiles such as elevated succinate or lactate levels [98]; and the “High Fibrosis Risk Phenotype” identified by imaging features such as COMP or DECT urate volume [108,133,134]. Furthermore, adaptive clinical trial designs, exemplified by umbrella trials, can simultaneously test multiple targeted therapies for different molecular subtypes within a single master protocol. Treatment response can be dynamically assessed using biomarkers, allowing for the flexible reassignment of patients between treatment arms [134,135]. Endpoint selection also requires innovation. In addition to traditional pain scores and serum uric acid levels, additional predictive endpoints must be incorporated to verify the effective modulation of the targeted pathways [13,108,120,136]. These should include imaging endpoints, notably the quantitative monitoring of urate deposition volume changes via DECT [128,137], and molecular endpoints, such as a confirmed reduction in H3K4me3 levels in macrophages [116] (Table 6).

In conclusion, through the deep integration of spatiotemporal multiomics and precision phenotyping, gout management is poised to enter a new era of predictability and targeted intervention [109,138,139].

### 4.3. Limitations of the Current Evidence and the Temporal Programming Model

While the “Gout Inflammation Time Programming” model provides a useful integrative framework, it is crucial to acknowledge the limitations of the current evidence and the gaps within this hypothesis. First, the model necessarily simplifies a highly heterogeneous disease. Clinical reality presents a spectrum where phases overlap, and not all patients progress linearly through the proposed stages. The biological basis of this resilience or rapid progression remains incompletely defined [113,114]. Second, several mechanistic links within the temporal cascade are supported by compelling but sometimes conflicting evidence. For instance, the dominant initial sensor cell (macrophage vs. mast cell), the net effect of NETosis (pro-inflammatory vs. pro-resolving) [9,11,38], and the consistency of specific metabolic markers like succinate across patient cohorts require further validation [30,32,35,36]. Third, key transitions in the program are still “black boxes.” The precise molecular signals that decisively tip the balance from the adaptive phase towards chronicity, or that initiate the epigenetic memory in stromal cells, are not yet fully elucidated [51,52,54]. Furthermore, while promising, technologies like digital twins are in their infancy, with significant challenges in data integration, model validation, and clinical utility remaining [102,106,107,109]. Acknowledging these limitations does not undermine the model’s utility but rather clarifies its purpose: to serve as a testable scaffold that explicitly highlights where future research—particularly the longitudinal, multi-omics studies and phenotype-driven trials advocated for in Section 4.2—is most urgently needed to refine our understanding of gout’s temporal pathophysiology.

## 5. Summary

This review comprehensively delineates the “Gout Inflammation Time Programming” model, framing gout as a temporally orchestrated pathological continuum from crystal triggering to tissue remodeling (Supplementary Table S1). Through the Gout-STAT™ framework, we conceptualize disease progression into three dynamically linked stages: the acute “perception” phase (0–24 h), characterized by MSU crystal recognition, *NLRP3* inflammasome activation, and neutrophil-driven inflammation [2,3,4,8] (Figure 1);the critical “adaptation” phase (24–72 h), defined by immunometabolic reprogramming of macrophages and synovial fibroblasts (Figure 3), where resolution or chronicity is determined [9,113,140], and the chronic “tissue injury” phase (>72 h), driven by epigenetic memory and leading to irreversible osteoarticular destruction (Figure 4) [13,141,142,143].

We further propose a paradigm shift toward phase-specific precision interventions. These include the targeting of upstream triggers such as mitochondrial ROS and NETosis in the acute phase [4,140], the rectification of metabolic checkpoints such as succinate accumulation and impaired autophagy during transition [140], the use of epigenetic modulators and biologic agents for tissue protection in the chronic phase [97,113,143]. The integration of cutting-edge technologies—such as intelligent responsive drug delivery systems [2,9,92] and digital twin joint models that leverage multiomics data [13,14,31,97]—is emphasized as a pivotal strategy for achieving spatiotemporally precise management.

To realize this vision, future research must prioritize actionable strategies in three interconnected domains. First, in mechanistic discovery, dynamic spatiotemporal multi-omics studies—employing serial single-cell RNA-sequencing and spatial transcriptomics on synovial fluid and tissue sampled across a flare timeline—are needed to construct a definitive ‘Inflammatory Atlas’ and identify the molecular tipping points between resolution and chronicity. Second, clinical translation requires a shift to precision trial design, enrolling patients based on molecular phenotypes (e.g., ‘Metabolic Dysregulation’ or ‘High Fibrosis Risk’, as proposed in Table 5) within adaptive master protocols (e.g., umbrella trials) to efficiently test matched targeted therapies. Third, a concerted effort is needed for biomarker validation, moving beyond static urate measurements to dynamically quantify target engagement (e.g., reduced H3K4me3 in macrophages) and treatment response (e.g., change in DECT urate volume) in clinical trials.

Ultimately, by executing these focused research priorities—decoding the molecular clock, validating precision stratification, and deploying intelligent therapies—we can transition gout management from a reactive model to a predictive, preventive, and personalized 4P medicine approach, fundamentally rewriting the disease trajectory for patients worldwide.

Despite this refined model, several mysteries persist, including the basis of disease resilience [2,97,113] and the precise mechanisms of the gut-joint axis [12,144]. Future research must leverage spatiotemporal multiomics integration and precision phenotyping in clinical trials to transition gout management from a reactive posture to a predictive, preventive, and personalized 4P medicine approach [22,29,145]. Ultimately, by decoding this intrinsic molecular clock and its phase-specific mechanisms, the Gout-STAT™ model provides the necessary framework to transition gout management from a reactive, symptom-driven model to a predictive, preventive, and personalized 4P medicine approach, fundamentally rewriting the disease trajectory for patients worldwide.

## Figures and Tables

**Table 2:** Targeted interventions for acute gout phase (<24 h).

Target	Intervention	Mechanism	Example Agent
SYK Kinase	Inhibitor	Blocks proximal signal transduction following crystal recognition	Fostamatinib
Mitochondrial ROS	Scavenger	Inhibits NLRP3 activation	MitoQ
NETosis	PAD4 inhibitor	Prevents histone citrullination	GSK484
IL-1β	Inhibitor delivery	Localized cytokine blockade	ROS-hydrogel/IL-1β inhibitor

This table summarizes emerging targeted therapies for the acute gout phase, highlighting their mechanisms, example agents, and current development stages, illustrating the move beyond conventional anti-inflammatory drugs.

**Table 3:** Metabolic and autophagic interventions during the transition phase (24–72 h).

Target	Intervention	Mechanism	Example Agent
Macrophage Glycolysis	AMPK activator	Shifts M1 → M2 metabolism	Metformin
Succinate/SUCNR1	Antagonist	Blocks inflammatory memory	SUCNR1 antagonist
Autophagy Flux	Inducer	Clears p62, inhibits mTORC1/fibrosis	Tat-Beclin1 peptide

This table outlines intervention strategies targeting macrophage metabolism and autophagy during the critical transition phase, aiming to promote inflammation resolution and prevent chronicity.

**Table 4:** Tissue-protective strategies for chronic gout (>72 h).

Strategy Type	Target	Intervention	Mechanism	Example Agent
Epigenetic Modulation	BET proteins	Inhibitor	Resets proinflammatory chromatin	JQ1
Stem Cell Protection	Wnt5a signaling	Monoclonal antibody	Blocks ectopic osteogenesis	Anti-Wnt5a mAb
Urate Excretion	*ABCG2* pathway	Corrective therapy	Enhances uric acid clearance	Under development

This table presents advanced strategies for chronic gout focusing on epigenetic modulation and stem cell protection to counteract established tissue memory and prevent structural joint damage.

**Table 6:** Proposed molecular phenotypes for precision enrollment in gout clinical trials.

Phenotype	Defining Biomarkers/Criteria	Potential Targeted Therapy
Metabolic Dysregulation	Elevated succinate/lactate; dysregulated nicotinamide metabolism	SUCNR1 antagonists, AMPK activators
High Fibrosis Risk	High COMP; DECT urate volume; mTORC1 activation	Autophagy inducers, anti-fibrotics
Epigenetic Dysregulation	High H3K4me3/H3K27ac; UMOD hypermethylation	BET inhibitors, HDAC inhibitors
Acute Inflammatory	High IL-1β, IL-6, neutrophil count	NLRP3 inhibitors, NETosis blockers

This table proposes molecular phenotypes for refining patient enrollment in clinical trials, facilitating the testing of targeted therapies in biologically relevant subgroups to improve trial success rates.

## Data Availability

No new data were created or analyzed in this study.

## Supplementary Materials

The following supporting information can be downloaded at: https://www.mdpi.com/article/10.3390/ijms27031523/s1.

## Abbreviations

The following abbreviations are used in this manuscript:

MSU	monosodium urate
NLRP3	NOD-like receptor thermal protein domain associated protein 3
ROS	reactive oxygen species
IL-1β	interleukin-1β
NETs	neutrophil extracellular traps
TLR4	Toll-like receptors 4
MYD88	Myeloid Differentiation Primary Response 88
ASICs	acid-sensing ion channels
SYK	Spleen tyrosine kinase
MRGPRX2	Mas-related G protein-coupled receptor member X2
STING	stimulator of interferon genes
ASC	apoptosis-associated speck-like protein containing a CARD
NETosis	neutrophil extracellular trap
MPO-HOCl	myeloperoxidase-hypochlorous acid
NAD	Nicotinamide Adenine Dinucleotide
SIRT1	silent information regulator 1
NF-κB	nuclear factor kappa-B
HDACs	histone deacetylases
CD39	Ectonucleoside triphosphate diphosphohydrolase-1
FLSs	Fibroblast-like synoviocytes
SQSTM1/p62	Sequestosome 1
mTORC1	Mechanistic Target of Rapamycin Complex 1
GAS	gout activity score
HDL-C	High density lipoprotein cholesterol
NUMB	NUMB endocytic adaptor protein
ABCG2	ATP binding cassette subfamily G member 2 Gene
TNF-α	tumor necrosis factor-α
IL-6	interleukin-6
H3K4me3	Tri-methylation of lysine 4 on histone H3
H3K27ac	H3 K27 acetylation
ox-LDL	oxidized low-density lipoprotein
hs-CRP	high-sensitivity C-reactive protein
TADs	topologically associating domains
NFIL3	nuclear factor, interleukin 3 regulated Gene
REDD1	Regulated in Development and DNA Damage Response 1
mTOR	mammalian target of rapamycin
MRI	magnetic resonance imaging
RANKL	Tumor necrosis factor ligand superfamily member 11
RANK	Tumor necrosis factor receptor superfamily member 11A
OPG	osteoclastogenesis inhibitory factor
BMP-2	Bone Morphogenetic Protein 2
DECT	Dual Energy Computed Tomography
WNT5A	Wnt family member 5A Gene
TGF-β	Transforming growth factor beta
T2T	treat-to-target
ROS-NLRP3	reactive oxygen species- NOD-like receptor thermal protein domain associated protein 3
MitoQ	Mitoquinone mesylate
PAD4	peptidylarginine deiminase 4
GSK484	peptidyl arginine deiminase 4 inhibitors
AMPK	Adenosine 5′-monophosphate (AMP)-activated protein kinase
MPC	mitochondrial pyruvate carrier
MLT-MLP	macrophage membrane-coated melatonin-loaded liposomes
SUCNR1	succinate receptor
BET	Bromodomain and extraterminal
JQ1	BET protein inhibitor
UMOD	uromodulin
CT	computed tomography
CARD8	caspase recruitment domain family member 8 Gene
TNF	tumor necrosis factor domains
SCFAs	short-chain fatty acids
TGF-β1	Transforming Growth Factor β1
COMP	artilage oligomeric matrix protein
